# Occupational Asbestos Exposure and Kidney Cancer: Systematic Review and Meta-analysis of Cohort Studies

**DOI:** 10.1093/annweh/wxaa114

**Published:** 2020-12-26

**Authors:** Chris C Y Pang, Kevin Phan, Md Nazmul Karim, Afsana Afroz, Matthew Winter, Deborah C Glass

**Affiliations:** 1 Monash Centre for Occupational and Environmental Health, Monash University, Melbourne, VIC, Australia; 2 Department of Surgery, Liverpool Hospital, Liverpool, NSW, Australia; 3 School of Public Health and Preventive Medicine, Monash University, Melbourne, VIC, Australia; 4 Department of Urology, Royal North Shore Hospital, NSW, Australia

**Keywords:** asbestos, kidney cancer, meta-analysis, occupational exposure, systematic review

## Abstract

**Objectives:**

An estimated 125 million workers are exposed to asbestos worldwide. Asbestos is classified by the International Agency for Research on Cancer as a Group 1 carcinogen. The association between occupational asbestos exposure and kidney cancer is not well established however. This study aimed to determine the mortality and incidence of kidney cancer in workers who have been exposed to asbestos. We performed a systematic review and meta-analysis to evaluate the association between occupational asbestos exposure and kidney cancer.

**Methods:**

Medline, EMBASE, and Web of Science were searched according to the Preferred Reporting Items for Systematic Reviews and Meta-Analyses (PRISMA) guidelines for articles on occupational asbestos exposure and kidney cancer. The studies reported the standardized mortality ratio (SMR) or standardized incidence ratio (SIR) of kidney cancer in workers exposed to asbestos. SMRs or SIRs with its 95% confidence interval (CI) were pooled using a fixed-effect model.

**Results:**

Forty-nine cohort studies involving 335 492 workers were selected for analysis. These studies included 468 kidney cancer deaths and 160 incident cases. The overall pooled-SMR of kidney cancer was 0.95 (95% CI: 0.86–1.05), with no significant heterogeneity (*P*_*Q*_ = 0.09, *I*^2^ = 24.87%). The overall pooled-SIR of kidney cancer was 0.95 (95% CI: 0.79–1.11), with no significant heterogeneity (*P*_*Q*_ = 0.68, *I*^2^ = 0.00%). Subgroup analysis did not find any increased association with occupational asbestos exposure. There was no evidence of publication bias with Egger’s test *P* values of 0.08 for mortality studies and 0.99 for incidence studies.

**Conclusions:**

This systematic review and meta-analysis did not show evidence of association between occupational asbestos exposure and kidney cancer mortality or incidence.

**What’s important about this paper**

This systematic review and meta-analysis was necessary as there remains uncertainty about whether asbestos causes kidney cancer. This meta-analysis has the largest population of asbestos workers to date (335 492 participants) with 468 kidney cancer deaths and 160 incident kidney cancer cases. Mortality and incidence of kidney cancer were not found to be elevated overall, or among subgroups with occupational asbestos exposure. However, weaknesses in the individual epidemiological studies may limit the ability to identify associations, and longer follow-up may be required to detect kidney cancer risk.

## Introduction

The World Health Organization estimates that there are about 125 million people exposed to asbestos in the workplace globally (Burki, 2009). Work-related asbestos exposure is associated with about 233 000 deaths annually (Furaya *et al.*, 2018). Asbestos is still used worldwide with more than 2 million tons consumed in 2018 (Furaya *et al.*, 2018).

The 2012 International Agency for Research on Cancer Monograph 100C (IARC, 2012) states that there is sufficient evidence that asbestos can cause mesothelioma and cancers of the lung and ovary. There is emerging research that suggests that asbestos exposure may lead to laryngeal cancer (Peng *et al.*, 2016), colorectal cancer (Kwak *et al.*, 2019), and prostate cancer (Peng *et al.*, 2019).

Renal cell cancer represents about 85% of kidney cancers, and renal pelvis cancer comprising the rest of kidney cancer cases (Lipworth *et al.*, 2009). The mean age at diagnosis for renal cell cancer is in the early 60s, and in the late 60s for renal pelvis cancer (Lipworth *et al.*, 2009). The 5-year survival rate for localized renal cell cancer cases is about 90% (Lipworth *et al.*, 2009). Kidney cancer has been associated with risk factors such as smoking, obesity, and hypertension. However, the relationship between asbestos exposure and development of kidney cancer is not well understood.

There is biological plausibility that asbestos may lead to kidney cancer. Animal studies have demonstrated that ingested or inhaled asbestos may pass through the bronchiolar epithelium and gastrointestinal mucosa and migrate to various sites in the body such as the kidneys and induce carcinogenesis (Kanazawa *et al.*, 1970; Cook and Olson, 1979; Guillemin *et al.*, 1989). Kanazawa *et al.* (1970) injected asbestos fibres subcutaneously into mice, and found that the fibres migrated from the site of injection and disseminated into the bloodstream and entered the kidneys.

A meta-analysis by Sali and Boffetta (2000) conducted on 37 cohort studies published before 1999 did not show an association between occupational asbestos exposure and kidney cancer [pooled-standardized mortality ratio (SMR) of 1.1 (95% confidence interval (CI): 0.9–1.3) and pooled-standardized incidence ratio (SIR) of 1.0 (95% CI: 0.7–1.3)].

There have been 19 cohort studies published since the previous meta-analysis. This meta-analysis included 468 kidney cancer deaths and 160 incident kidney cancer cases.

In recent years, cohort studies have demonstrated conflicting results. A British cohort study by Harding *et al.* (2009) involving 98 117 asbestos workers showed a statistically significant positive association between occupational asbestos exposure and the development of kidney cancer [SMR = 1.52 (95% CI: 1.26–1.83)]. An Italian cohort study by Ferrante *et al.* (2017) involving 46 060 workers did not show an increased kidney cancer risk [SMR = 0.98 (95% CI: 0.83–1.14)].

An updated meta-analysis is appropriate to analyse all published cohort studies. If there was a true association found between occupational asbestos exposure and the development of kidney cancer, it may influence the health surveillance protocols for workers exposed to asbestos. It may also affect asbestos-related compensation claims.

The aim of this study is to perform a systematic review and meta-analysis of cohort studies to investigate the association between occupational asbestos exposure and kidney cancer risk.

## Methods

**Figure 1.**
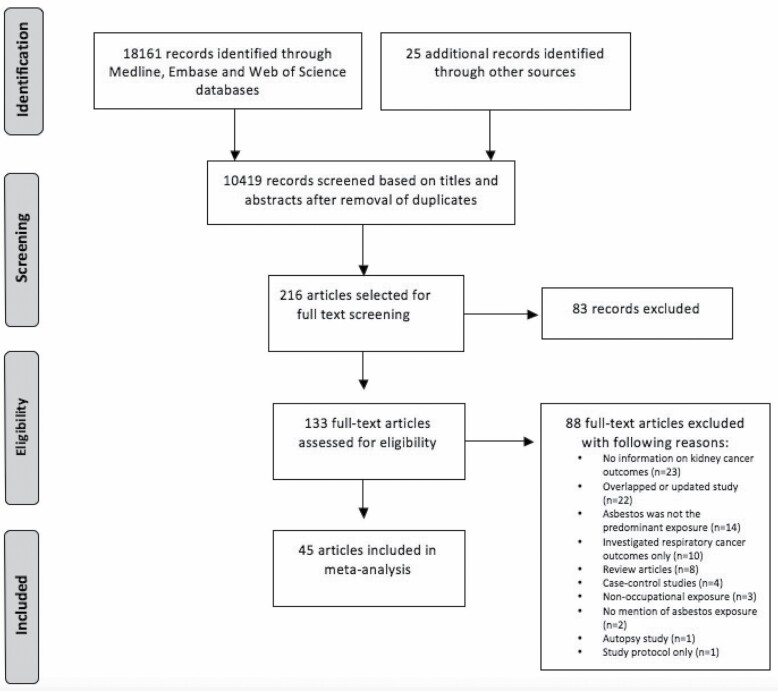
PRISMA flow diagram of the meta-analysis.

The systematic review and meta-analysis were reported according to the Preferred Reporting Items for Systematic Reviews and Meta-Analyses (PRISMA) guidelines (Liberati *et al.*, 2009). Fig. 1 shows the PRISMA flow diagram of the identification and screening of studies for inclusion in the meta-analysis.

Two reviewers (C.P. and K.P.) independently reviewed all the articles and extracted the data.

We searched on Medline, EMBASE, and Web of Science up to 31 December 2019. Search terms were the following:

‘Asbestos’, ‘Chrysotile’, ‘Amphiboles’, ‘Occupational exposure’, ‘Work exposure’, ‘Cancer’, ‘Kidney cancer’, ‘Renal cancer’, ‘Renal cell carcinoma’, ‘Neoplasm’, ‘Malignancy’, ‘Mortality’, and ‘Cohort study’. The reference lists of relevant publications were also reviewed manually to identify additional studies.

Selection of studies was based on a clearly defined set of inclusion and exclusion criteria. If studies involving the same cohort had been published previously, only the most recent study was included.

To be eligible for inclusion, studies had to meet the following criteria:

1. Cohort study with published data.
2. Contained information to estimate the relation between asbestos exposure and kidney cancer risk (effect size) in terms of relative risk, hazard ratio, SMR, or SIR.
3. Included workers with predominant exposure to asbestos, such as asbestos cement, textile, and mining workers.

The studies were excluded if they met the following criteria:

1. Overlapping articles or duplicate data.
2. Animal studies.
3. Meta-analyses or review articles.
4. No kidney cancer outcomes.
5. Studies which included asbestos exposure that did not occur in the workplace.

Data containing name of first author, publication year, geographic location of cohort, industry type, asbestos type, exposure assessment, period of employment, cohort size, follow-up period, total person-years of observation, SMR or SIR, and 95% CIs for kidney cancer, observed and expected kidney cancer cases or deaths were identified. Data were extracted and transferred to Microsoft Excel by two authors (C.P. and K.P.) for each eligible study. All disagreements arising from this process were resolved by discussion.

C.P. and K.P. assessed the risk bias of each eligible study using the Newcastle–Ottawa Scale (NOS) tool (Lo *et al.*, 2014). The tool utilizes a ‘star system’ in which a study is judged on three broad perspectives: the selection of the study groups; the comparability of the groups; and the ascertainment of the exposure of interest for cohort studies.

A total of 45 articles representing 49 cohort studies met the criteria for inclusion in the meta-analysis. Cohort studies that reported SMR were termed ‘Mortality studies’ and studies that reported SIR or hazard ratio were termed ‘Incidence studies’.

Analysis was done separately for the studies that reported SMRs, and those that reported SIRs. One study reporting the hazard ratio was combined with the studies reporting SIRs.

The overall SMR/SIR estimates and corresponding 95% CIs were calculated using fixed-effects and random-effects models (Mantel and Haenszel, 1959). Between-study heterogeneity was assessed using the *Q* and *I*^2^ statistics (Higgins and Thompson, 2002). This study considered a value of *I*^2^ >25% to indicate substantial heterogeneity (Higgins and Thompson, 2002). Cochran *Q* test was also used to assess heterogeneity, with a *P*_*Q*_ < 0.1 considered statistically significant for heterogeneity (Higgins and Thompson, 2002).

Subgroup analysis using the fixed-effects model was carried out for the following covariates: asbestos type, industry type, geographic location of cohort, cohort size, follow-up period, total person-years follow-up, and NOS rating. Subgroup analyses were done separately for mortality studies and incidence studies.

C.P. and K.P. reviewed the quality of exposure assessment of these studies using the framework proposed by Lenters *et al.* (2011). The studies were assessed on the following five exposure assessment aspects: documentation, cumulative exposure ratio, conversion factor, coverage of exposure data, and job histories (Lenters *et al.*, 2011). Additional analyses were carried examining the association between kidney cancer and asbestos exposure for the studies with adequate exposure assessment quality.

Sensitivity analysis was conducted using the ‘leave-one-out’ method to ascertain the influence of any single study on the overall result (Viechtbauer and Cheung, 2010).

Publication bias was assessed by visual inspection of Begg’s funnel plots and Egger’s regression test (Sterne and Egger, 2001). Statistical significance was defined as *P* value <0.05 for all analyses except for heterogeneity.

The meta-analysis was completed with STATA software Version 16.0 (StataCorp, 2019). Ethics approval was not required for this systematic review and meta-analysis.

## Results

The titles and abstracts for 10 419 papers were screened as shown in Fig. 1. The full text for 133 articles was eventually assessed for eligibility for inclusion, and 88 of these articles were excluded. A total of 49 studies were included in this meta-analysis.

These studies were published between 1979 and 2018. The cohort sizes ranged from 541 to 98 117. The mortality studies included 276 214 workers and the incidence studies included 59 278 workers. Total person-years of follow-up were available for 26 mortality studies (total: 4 909 904 person-years) and 9 incidence studies (total: 704 877 person-years). Ninety-two percent of the total cohort participants were male workers.

Out of 49 studies, 38 studies reported SMR, 10 studies reported SIR, and 1 study reported the hazard ratio. Studies were conducted in Europe, North America, Asia, and Australia. The main industries represented by the studies included the mining, textile, cement, and insulation industries. Other asbestos workers included those working in the shipyards, ship breaking industry, rail industry, asbestos plants, and friction material manufacturing.

The follow-up period for these studies ranged from 10 to 69 years for the mortality studies (mean: 35 years; standard deviation: 14) and ranged from 8 to 47 years for the incidence studies (mean: 30.7 years; standard deviation: 14.7).

There was a total of 468 kidney cancer deaths among the 38 mortality studies. SMR estimates reported by the individual studies ranged from 0 to 5.00.

There was a total of 160 incident kidney cancer cases among the 11 incidence studies. SIR estimates reported by the individual studies ranged from 0.48 to 1.85.

Authors collected smoking data for 22 of the mortality studies, and 8 of the incidence studies.

The main characteristics of the included mortality and incidence studies are shown in Supplementary Tables S1 and S2 (available at *Annals of Work Exposures and Health* online edition). The reasons for exclusion of studies are shown in Supplementary Table S3 (available at *Annals of Work Exposures and Health* online edition).

**Figure 2.**
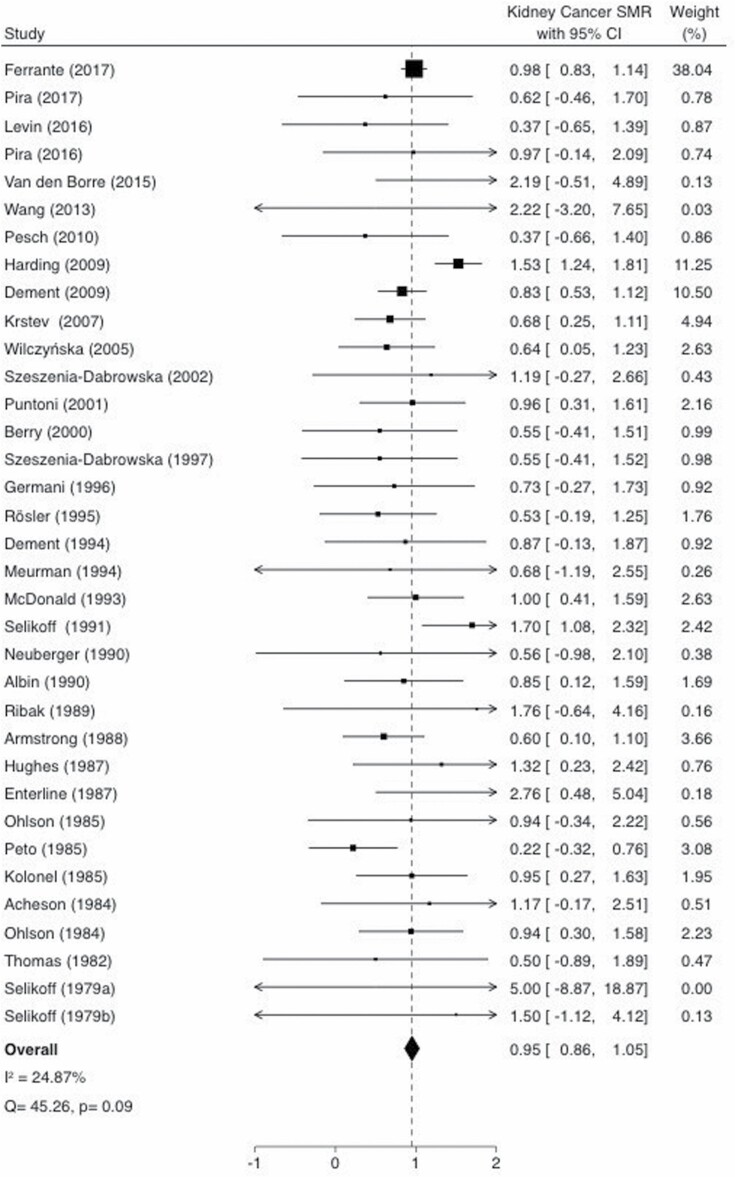
SMR estimates and 95% CIs of kidney cancer associated with occupational asbestos exposure. Weights are from fixed-effects analysis. Study-specific SMRs are shown as squares, with the horizontal lines representing the 95% CIs for the study-specific SMRs. The area of the squares represents weight of the individual study. The pooled-SMR is shown as a diamond. The middle of the diamond corresponds to the pooled-SMR, and the width of the diamond represents the 95% CI. The vertical dashed line provides a visual comparison of the pooled-SMR with the corresponding study-specific SMRs. *I*^2^, *I*^2^ statistic; *P*, *P* value for Cochran *Q* test; *Q*, Cochran *Q* test.

The pooled-SMR based on the fixed-effects model for kidney cancer among workers with occupational asbestos exposure was 0.95 (95% CI: 0.86–1.05), which is shown as a Forest Plot in Fig. 2. There was no evidence of significant heterogeneity among the studies (*Q* = 45.26, *P*_*Q*_ = 0.09, *I*^2^ = 24.87).

The random-effects model yielded a similar effect size of 0.89 (95% CI: 0.73–1.05).

Three mortality studies with zero observed and/or expected events were removed from the meta-analysis (Acheson *et al.*, 1982; Gardner *et al.*, 1986). These three mortality studies represented 1.3% of the total cohort size, and it should not affect the meta-analysis.

**Figure 3.**
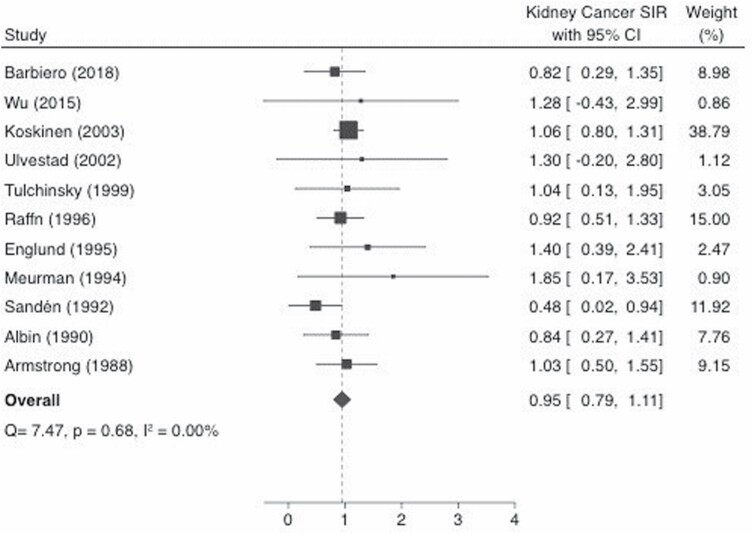
SIR estimates and 95% CIs of kidney cancer associated with occupational asbestos exposure. Weights are from fixed-effects analysis. Study-specific SIRs are shown as squares, with the horizontal lines representing the 95% CIs for the study-specific SIRs. The area of the squares represents weight of the individual study. The pooled-SIR is shown as a diamond. The middle of the diamond corresponds to the pooled-SIR, and the width of the diamond represents the 95% CI. The vertical dashed line provides a visual comparison of the pooled-SIR with the corresponding study-specific SIRs. *I*^2^, *I*^2^ statistic; *P*, *P* value for Cochran *Q* test; *Q*, Cochran *Q* test.

The pooled-SIR based on the fixed-effects model for kidney cancer was 0.95 (95% CI: 0.79–1.11), which is shown as a Forest Plot in Fig. 3. There was no evidence of significant heterogeneity among the studies (*Q* = 7.47, *P*_*Q*_ = 0.68, *I*^2^ = 0.00%). The random-effects model yielded a similar effect size of 0.95 (95% CI: 0.78–1.11).

There was no association between asbestos type and kidney cancer. The SMRs for Chrysotile and Amphibole exposure were 0.73 (95% CI: 0.46–1.01) and 0.65 (95% CI: 0.25–1.06), respectively. Workers who were exposed to undefined asbestos fibres had an SMR of 1.01 (95% CI: 0.90–1.11).

Table 1 shows the fixed-effects model-based pooled estimates and 95% CIs of kidney cancer outcomes by study characteristics for mortality studies.

**Table 1.** Fixed-effects model-based pooled-SMR estimates and 95% CIs of kidney cancer deaths associated with asbestos exposure by mortality study characteristics.

Study characteristics	No. of studies	Pooled-SMR (95% CI)	*Q*	*P*_*Q*_	*I* ^2^ (%)
All	35	0.95 (0.86 to 1.05)	45.26	0.09	24.87
Type of asbestos					
Chrysotile	11	0.73 (0.46 to 1.01)	6.23	0.8	0.0
Amphibole	5	0.65 (0.25 to 1.06)	1.73	0.79	0.0
Undefined	19	1.01 (0.90 to 1.11)	31.81	0.02	43.4
Industry type					
Mining	4	0.75 (0.40 to 1.10)	1.1	0.78	0.0
Textile	4	0.47 (0.04 to 0.91)	2.6	0.46	0.0
Cement	6	0.82 (0.38 to 1.25)	1.45	0.92	0.0
Mixed	7	1.07 (0.94 to 1.20)	16.4	0.01	63.4
Others	14	0.89 (0.71 to 1.06)	13.78	0.39	5.7
Cohort size					
<5000	26	0.71 (0.53 to 0.89)	13.02	0.98	0.0
≥5000	9	1.05 (0.93 to 1.16)	22.6	0.00	64.6
Follow-up period					
<25 years	8	0.89 (0.68 to 1.09)	10.6	0.16	33.8
≥25 years	27	0.97 (0.86 to 1.08)	34.15	0.13	23.9
Person-years follow-up					
<100 000 person-years	15	0.88 (0.56 to 1.20)	5.84	0.97	0.0
≥100 000 person-years	9	1.01 (0.90 to 1.12)	25.66	0.00	68.8
No data	11	0.69 (0.44 to 0.94)	8.2	0.62	0.0
NOS rating					
Good	30	0.94 (0.84 to 1.03)	37.7	0.13	23.1
Fair	3	1.49 (0.98 to 2.00)	1.44	0.49	0.0
Poor	2	0.59 (−0.36 to 1.53)	1.09	0.3	8.1
Geographic location					
Europe	21	0.98 (0.86 to 1.09)	30.03	0.07	33.4
North America	12	0.93 (0.74 to 1.12)	12.9	0.30	14.7
Asia	1	2.22 (−3.21 to 7.65)	0	NA	NA
Australia	1	0.60 (0.10 to 1.10)	0	NA	NA

*I* ^2^, *I*^2^ statistic; NA, not applicable; *P*_*Q*_, *P* value for Cochran *Q* test; *Q*, Cochran *Q* test.

There was no significant association shown between workers in the mining, cement, or mixed industries, and kidney cancer SMRs. The kidney cancer SMR of those who worked in the textile industry had a marginally statistically significant pooled-SMR of 0.47 (95% CI: 0.04–0.91).

There was no statistically significantly association between the kidney cancer SMRs in the various subgroups and cohort size, follow-up period, person-years follow-up, NOS rating and geographic location.

Table 2 shows the fixed-effects model-based pooled estimates and 95% CIs of kidney cancer outcomes by study characteristics for incidence studies.

**Table 2.** Fixed-effects model-based pooled-SIR estimates and 95% CIs of kidney cancer cases associated with asbestos exposure by incidence study characteristics.

Study characteristics	No. of studies	Pooled-SIR (95% CI)	*Q*	*P*_*Q*_	*I* ^2^ (%)
All	11	0.95 (0.79–1.11)	7.47	0.68	0.0
Type of asbestos					
Chrysotile	3	0.93 (0.47–1.39)	0.39	0.83	0.0
Amphibole	4	0.83 (0.57–1.09)	4.39	0.22	31.7
Undefined	4	1.04 (0.82–1.26)	1.25	0.74	0.0
Industry type					
Mining	2	1.10 (0.60–1.60)	0.84	0.36	0.0
Cement	4	0.93 (0.62–1.23)	0.39	0.94	0.0
Mixed	3	1.03 (0.81–1.26)	1.17	0.56	0.0
Others	2	0.53 (0.09–0.98)	0.78	0.38	0.0
Cohort size					
<5000	8	0.82 (0.56–1.08)	5.72	0.57	0.0
≥5000	3	1.02 (0.82–1.22)	0.32	0.85	0.0
Follow-up period					
<25 years	5	0.93 (0.73–1.13)	5.83	0.21	31.4
≥25 years	6	0.97 (0.71–1.24)	1.57	0.90	0.0
Person-years follow-up					
<100 000 person-years	5	0.74 (0.45–1.03)	3.66	0.46	0.0
≥100 000 person-years	4	1.03 (0.83–1.23)	0.41	0.94	0.0
No data	2	1.20 (0.53–1.88)	0.27	0.60	0.0
NOS rating					
Good	11	0.95 (0.79–1.11)	7.47	0.68	0.0
Geographic location					
Europe	8	0.93 (0.76–1.10)	7.16	0.41	2.3
Asia	2	1.10 (0.29–1.90)	0.06	0.81	0.0
Australia	1	1.03 (0.51–1.56)	0	NA	NA

*I* ^2^, *I*^2^ statistic; NA, not applicable; *P*_*Q*_, *P* value for Cochran *Q* test; *Q*, Cochran *Q* test.

There was no association between types of asbestos fibre exposure and kidney cancer SIRs. Workers who were exposed to chrysotile and amphibole fibres had SIRs of 0.93 (95% CI: 0.47–1.4) and 0.83 (95% CI: 0.57–1.09), respectively.

There was no association between working in the mining, cement, and mixed industries, and kidney cancer SIR, with SIRs of 1.10 (95% CI: 0.60–1.60), 0.93 (95% CI: 0.62–1.23), and 1.03 (95% CI: 0.81–1.26), respectively. In comparison, workers in two studies representing the shipyard and ship breaking industries had a marginally lower SIR of 0.53 (95% CI: 0.09–0.98).

Other study characteristics such as cohort size, follow-up period, person-years follow-up, and geographic location did not have any statistically significant association with kidney cancer incidence.

There were six studies that provided exposure assessment data specific to kidney cancer outcomes, including five mortality studies and one incidence study. The exposure assessment across all six studies was of varying quality, graded according to the characteristics proposed by Lenters *et al.* (2011). The mortality studies by Berry *et al.* (2000), McDonald *et al.* (1993), Hughes *et al.* (1987), and Peto *et al.* (1985) described adequate exposure assessment processes, with the studies each meeting three, five, four, and five criteria, respectively. The mortality study by Selikoff *et al.* (1979) only met one criterion. The sole incidence study by Sandén *et al.* (1992) met three of the five criteria (see Supplementary Table S4, available at *Annals of Work Exposures and Health* online edition).

The pooled-SMR for the four mortality studies with adequate exposure assessment data was 0.65 (95% CI: 0.30–1.00) as shown in Supplementary Fig. S5 (available at *Annals of Work Exposures and Health* online edition). This showed that studies with good exposure assessment specific to kidney cancer outcomes, also did not demonstrate an association between occupational asbestos exposure and kidney cancer outcomes.

Sensitivity analysis showed that the pooled-SMRs were relatively robust to the exclusion of any one study from the overall meta-analysis and did not change by more than 10% (see Supplementary Fig. S6, available at *Annals of Work Exposures and Health* online edition). The pooled-SMR was marginally statistically significant after leaving out the Harding study [SMR: 0.88 (95% CI: 0.78–0.98)] (Harding *et al.*, 2009). Overall heterogeneity was reduced after the Harding study was removed from the pooled-SMR (*P*_*Q*_ = 0.74, *I*^2^ = 0.00%).

Sensitivity analysis of the incidence studies showed that the pooled-SIRs did not significantly change after excluding studies one by one. This suggested stability to the pooled-SIR results (see Supplementary Fig. S7, available at *Annals of Work Exposures and Health* online edition).

**Figure 4.**
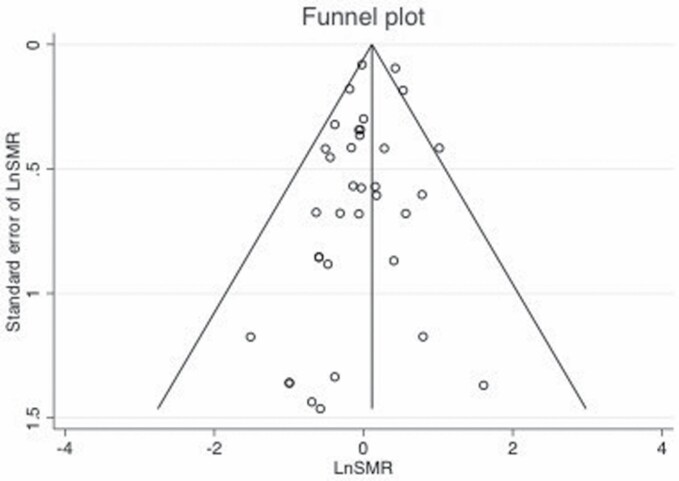
Begg’s funnel plot for evaluating publication bias showing the natural logarithm of kidney cancer SMR (LnSMR) plotted against the standard error of the LnSMR.

**Figure 5.**
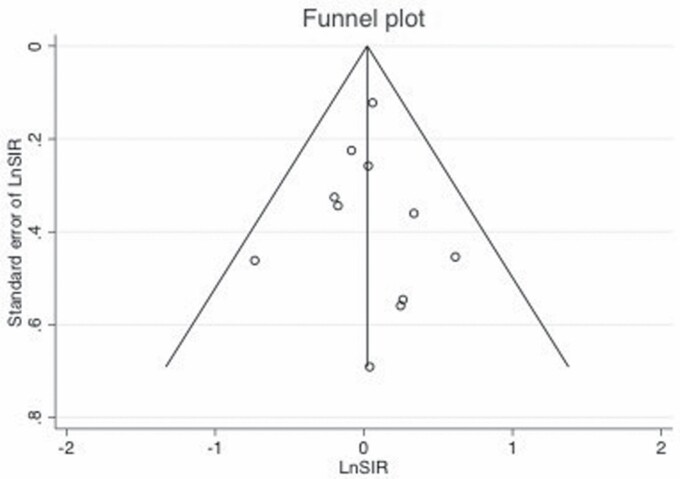
Begg’s funnel plot for evaluating publication bias showing the natural logarithm of kidney cancer SIR (LnSIR) plotted against the standard error of the LnSIR.

Begg’s funnel plots shown in Figs 4 and 5 did not demonstrate significant asymmetry.

Additionally, Egger’s regression test did not show evidence of publication bias among the mortality studies (Egger, *P* = 0.08) nor among the incidence studies (Egger, *P* = 0.99).

## Discussion

This systematic review and meta-analysis assessed the association between occupational asbestos exposure and kidney cancer based on 49 cohort studies.

The results based on the fixed-effects model showed no statistically significant difference in the estimates of the pooled-SMR (0.95; 95% CI: 0.86–1.05) and pooled-SIR (0.95; 95% CI: 0.79–1.11). For the incidence studies, SIRs from 10 studies and a hazard ratio from 1 study were pooled. The SIR and hazard ratio are generated through multivariable regression models and reflect the risk of the outcome in prospective data, hence combining the two is unlikely to produce heterogeneity.

There is a high survival rate among people with kidney cancer (Lipworth *et al.*, 2009). Workers in this study may have died of other causes such as lung cancer, leading to under-reporting of the number of kidney cancer deaths. The SIR which takes into account the number of new kidney cancer cases may be the better indicator of kidney cancer risk among these asbestos workers.

The workforce in these studies may have demonstrated the ‘healthy worker effect’ (McMichael, 1976), but this effect is limited as kidney cancer is usually associated with advanced age (Ji *et al.*, 2005). It is also possible that some of the results were due to multiple comparisons in studies that examined many cancer sites. The multiple comparisons phenomenon occurs where a small number of statistically significant results could be expected to occur simply by chance (McDonald, 2014).

The pooled estimates for larger cohort sizes (≥5000 workers) were higher compared with smaller cohort sizes (<5000 workers), with narrower CIs. This is consistent with increased precision of results with an increased sample size. Higher pooled estimates with an increased precision of the estimate size were also observed for longer person-years of follow-up (≥100 000 person-years) compared with shorter person-years of follow-up (<100 000 person-years), which is consistent with kidney cancer being a disease associated with advanced age.

Sensitivity analysis demonstrated that the Harding cohort contributed to the heterogeneity among the mortality studies, which was reduced after the study was removed from the meta-analysis (*P*_*Q*_ = 0.74, *I*^2^ = 0.00%) (Harding *et al.*, 2009). It was the only study among all 38 mortality studies that reported a statistically significant positive association with occupational asbestos exposure, with an SMR of 1.53 (95% CI: 1.26–1.83). Harding *et al.* (2009) reported that the kidney cancer SMR but not the proportional mortality rates was statistically significantly raised. Hence, they concluded that the observed kidney cancer deaths were due to confounding factors such as smoking rather than to asbestos exposure.

There was little evidence of significant heterogeneity in this meta-analysis. There was no evidence of publication bias among the studies.

The limitations of this meta-analysis include the lack of exposure data to allow the investigation of the dose–response relationship. Exposure assessment methods varied across the studies, and mostly analysed the dose–response effect for lung cancer or mesothelioma. Additionally, there was paucity of information about the cumulative exposure at the individual level.

Three mortality studies provided dose–exposure data in relation to kidney cancer (Peto *et al.*, 1985; McDonald *et al.*, 1993; Berry *et al.*, 2000). The three studies used different exposure categories, and this prohibited any meaningful analysis of occupational asbestos exposure levels in relation to kidney cancer risk.

Berry *et al.* (2000) reported an SMR of 0.55 (95% CI: 0.07–1.99) for workers with severe asbestos exposure for more than 2 years. McDonald *et al.* (1993) reported a non-significantly elevated SMR of 1.35 (95% CI: 0.50–2.95) for workers exposed to an average of 300 million or more particles of asbestos fibres per cubic foot per year. Peto *et al.* (1985) reported that for a group of male workers employed for more than 20 years, the SMR was 0.4 (95% CI: 0.01–2.23). A clear dose–response effect could not be confirmed based on these three studies.

The latent period between asbestos exposure and kidney cancer development is unknown (Peters *et al.*, 2018). Two mortality studies and one incidence study investigated the potential effect of latency on development of kidney cancer (Selikoff *et al*.,1979; Hughes *et al.*, 1987; Sandén *et al.*, 1992).

Hughes *et al.* (1987) reported a kidney cancer SMR of 1.32 (95% CI: 0.53–2.72) among workers 20 or more years since initial occupational asbestos exposure. Selikoff *et al.* (1979) reported 2 kidney cancer deaths for 498 insulation workers 35 years after onset of exposure and reported that the SMR for these workers was 5.56 (95% CI: 1.80–12.96). The study did not find any kidney cancer deaths for workers with less than 35 years after onset of exposure. Sandén *et al.* (1992) reported an SIR of 0.61 (95% CI: 0.13–1.8) for workers with at least 20 years since onset of heavy occupational asbestos exposure. There was no increasing trend of kidney cancer cases with a longer latent period. We could not formulate any conclusions about the latent period of kidney cancer from these three studies. It is possible that the latent period for the studies in this meta-analysis was insufficient for kidney cancers to develop. A longer follow-up period may be required to detect any potential kidney cancer outcomes.

Poor exposure assessment can result in a bias to the null (Copeland *et al.*, 1977), so we reviewed the exposure assessment quality and reanalysed only those with adequate exposure assessment. There were only six studies that provided exposure assessment data specific to kidney cancer outcomes, including five mortality studies and one incidence study as aforementioned (Selikoff *et al.*, 1979; Peto *et al.*, 1985; Hughes *et al.*, 1987; Sandén *et al.*, 1992; McDonald *et al.*, 1993; Berry *et al.*, 2000). The pooled-SMR for the four mortality studies with adequate exposure assessment quality did not demonstrate an association between occupational asbestos exposure and kidney cancer outcomes.

Furthermore, subgroup analysis according to the various NOS ratings of the individual studies did not show any association with kidney cancer outcomes. We concluded that neither the quality of exposure assessment nor the overall quality of the individual cohort studies affected the final result of this meta-analysis.

There were potential weaknesses in ascertaining the occupational history for workers in some studies which involved answering self-reporting questionnaires. This may have led to inaccurate and inconsistent description of their work duties and quantification of asbestos exposure. Furthermore, there was a lack of information about workers’ subsequent occupation after leaving the asbestos industry for which there may have been exposure to other chemical hazards.

The comparison population for the included studies was the general population from which the workers were derived. It is possible that the control population included asbestos exposed individuals, thus potentially causing a bias towards the null.

These asbestos exposed workers were at higher risk of developing other cancers such as lung cancer and mesothelioma with a relatively short lag time between disease manifestation and death compared with kidney cancer. This may have resulted in decreased detection of kidney cancer cases, which may potentially bias the overall results towards the null.

Risk of bias assessment was conducted using the NOS which has potential limitations. These limitations include subjectivity in interpreting the NOS criteria which may affect inter-rater reliability (Lo *et al.*, 2014). There was a high degree of agreement of the NOS rating between the reviewers, and any disagreement was resolved through discussion. Hence, this factor would not have impacted this study’s results.

## Conclusions

This meta-analysis showed a lack of association between occupational asbestos exposure and kidney cancer risk. The summary risk estimates for both the mortality and incidence studies were not elevated. There was no clear dose–response relationship. These results are consistent with the results from the previous meta-analysis by Sali and Boffetta (2000).

While there is biological plausibility from animal studies of occupational asbestos exposure causing kidney cancer, this meta-analysis does not provide evidence to support this association. The inherent weaknesses of the individual studies including the lack of exposure and dose–response data, prohibited the establishment of occupational asbestos exposure as a potential cause of kidney cancer.

Further research is required including characterization of occupational exposure levels at an individual level. Longer-term follow-up may also be required to detect any potential kidney cancer risk and improve the precision of outcome measures. Data about known confounders for kidney cancer such as smoking, and obesity should also be incorporated into future research.

## Supplementary Material

wxaa114_suppl_Supplementary_Tables_S1_S2_S3

wxaa114_suppl_Supplementary_Tables_S1_S2_S3_Review

wxaa114_suppl_Supplementary_Figures_S5_S6_S7
