# Supplementary material for: Occupational Asbestos Exposure and Kidney Cancer: Systematic Review and Meta-analysis of Cohort Studies
Source: Ann Work Expo Health. 2020 Dec 26;65(3):255–65. doi: 10.1093/annweh/wxaa114 (PMC8062011; doi:10.1093/annweh/wxaa114)
Supplement: wxaa114_suppl_Supplementary_Tables_S1_S2_S3 [file wxaa114_suppl_supplementary_tables_s1_s2_s3.docx]

**Title:** Occupational asbestos exposure and kidney cancer: Systematic Review and Meta-analysis of Cohort Studies.

**Authors:** Chris CY Pang, Kevin Phan, Md Nazmul Karim, Afsana Afroz, Matthew Winter, Deborah C Glass

**Additional references for Supplementary Tables S1, S2 and S3**

Acheson ED, Gardner MU, Winter PD, Bennett C.(1984) Cancer in a factory using amosite asbestos. Int J Epidemiol;13: 3-10.

Albin M, Jakobsson K, Attewell R, Johansson L, Welinder H.(1990)Mortality and cancer morbidity in cohorts of asbestos cement workers and referents. Br J Ind Med;47: 602-610.

Alies-Patin AM, Valleron AJ.(1985) Mortality of workers in a French asbestos cement factory 1940-82. Br J Ind Med;42:219-225.

Armstrong BK, de Klerk NH, Musk AW, Hobbs MST.(1988) Mortality in miners and millers of crocidolite in Western Australia. Br J Ind Med;45: 5-13.

Barbiero F, Zanin T, Pisa F, *et al*. (2018) Cancer incidence in a cohort of asbestos-exposed workers undergoing health surveillance. Int Arch Occup Environ Health;91:831-841.

Barone-Adesi F, Ferrante D, Chellini E, *et al*.(2019) Role of asbestos clearance in explaining long-term risk of pleural and peritoneal cancer: a pooled analysis of cohort studies. Occup Environ Med;76:611-616.

Battista G, Belli S, Comba P, *et al*.(1999) Mortality due to asbestos-related causes among railway carriage construction and repair workers. Occup Med;49:536-539.

Beaumont JJ, Weiss NS.(1980) Mortality of welders, shipfitters, and other metal trades workers in boilermakers Local No. 104, AFL-CIO. Am J Epidemio;112:775-786.

Berry G. (1994) Mortality and cancer incidence of workers exposed to chrysotile asbestos in the friction-products industry. Ann Occup Hyg;38:539-546.

Blot WJ, Stone BJ, Fraumeni JF, *et al*.(1979) Cancer mortality in U.S. counties with shipyard industries during World War II. Environ Res;18:281-290.

Bunderson-Schelvan M, Pfau JC, Crouch R, *et al*.(2011) Nonpulmonary outcomes of asbestos exposure. J Toxicol Environ Health B Crit Rev;14:122-152.

Cheng WN, Kong J.(1992) A retrospective mortality cohort study of chrysotile asbestos products workers in Tianjin 1972-1987. Environ Res;59:271-278.

Clin B, Morlais F, Launoy G, *et al*.(2011) Cancer incidence within a cohort occupationally exposed to asbestos: a study of dose--response relationships. Occup Environ Med;68:832-836.

Courtice MN, Demers PA, Takaro TK, *et al*.(2011) Asbestos-related disease in Bangladeshi ship breakers: a pilot study. Int J Occup Environ Health;17:144-153.

DeBono N, Richardson D, Keil A, *et al*.(2019) Employment characteristics and cause-specific mortality at automotive electronics manufacturing plants in Huntsville, Alabama. Am J Ind Med; 62:296-308.

Delahunt B, Bethwaite PB, Necey JN.(1995) Occupational risk for renal cell carcinoma. A case-control study based on the New Zealand Cancer Registry. Br J Urol;75:578-582.

Dement J, Welch L, Haile E, *et al*.(2009) Mortality Among Sheet Metal Workers Participating in a Medical Screening Program. Am J Ind Med; 52:603-613.

Dement JM, Harris RL Jr, Symons MJ, *et al*.(1983) Exposures and mortality among chrysotile asbestos workers. Part II: mortality. Am J Ind Med;4:421-433.

Dement JM.(1991) Carcinogenicity of chrysotile asbestos: a case control study of textile workers. Cell Biol Toxicol;7:59-65.

Dement JM, Brown DP, Okun A.(1994) Follow-up study of chrysotile asbestos textile workers: cohort mortality and case-control analyses. Am J Ind Med; 26: 431-447.

Du L, Wang X, Wang M, *et al*.(2012)Analysis of mortality in chrysotile asbestos miners in China. J Huazhong Univ Sci Technolog Med Sci; 32:135-140.

Elmes PC, Simpson MJ.(1977) Insulation workers in Belfast. A further study of mortality due to asbestos exposure (1940-75). Br J Ind Med;34:174-180.

Englund A.(1995) Recent data on cancer due to asbestos in Sweden. Med Lav; 86: 435-439.

Enterline PE, Hartley J, Henderson V.(1987) Asbestos and cancer: a cohort followed up to death. Br J Ind Med;44: 396-401.

Ferrante D, Bertolotti M, Todesco A, *et al*.(2007)Cancer mortality and incidence of mesothelioma in a cohort of wives of asbestos workers in Casale Monferrato, Italy. Environ Health Perspect;115:1401-1405.

Finkelstein MM.(1989) Mortality rates among employees potentially exposed to chrysotile asbestos at two automotive parts factories. CMAJ; 141:125-130.

Finkelstein MM, Meisenkothen C.(2019) Malignant Mesothelioma Among Employees of a Connecticut Factory That Manufactured Friction Materials Using Chrysotile Asbestos: An Update. Ann Occup Hyg; 54:692-696.

Germani D, Belli S, Bruno C, *et al*.(1999)Cohort mortality study of women compensated for asbestosis in Italy. Am J Ind Med;36:129-134.

Germani D, Grignoli M, Belli S, *et al*.(1996) A mortality study of recipients of compensation for asbestosis in Italy (1980-1990). Med Lav;87: 371-385

Giaroli C, Belli S, Bruno C, *et al*.(1994) Mortality study of asbestos cement workers. Int Arch Occup Environ Health;66:7-11.

Gibbs AR, Gardner MJ, Pooley FD, *et al*.(1994) Fiber levels and disease in workers from a factory predominantly using amosite. Environ Health Perspect;102:261-263.

Giordano F, Dell'orco V, Fantini F, *et al*.(2012) Mortality in a cohort of cement workers in a plant of Central Italy. Int Arch Occup Environ Health;85:373-379.

Golka K, Wiese A, Assennato G, *et al*.(2004) Occupational exposure and urological cancer. World J Urol ;21:382-391.

Gun R, Pratt NL, Roder DM, *et al*.(2006) Asbestos-related cancers in refinery workers in the Australian petroleum industry. Arch Environ Occup Health;61:11-16.

Heck J, Charbotel B, Moore L, *et al*.(2010) Occupation and renal cell cancer in Central and Eastern Europe. Occup Environ Med; 67:47-53.

Hein MJ, Stayner LT, Lehman E, *et al*.(2007) Follow-up study of chrysotile textile workers: cohort mortality and exposure-response. Occup Environ Med;64:616-625.

Hodgson JT, Jones RD.(1986) Mortality of asbestos workers in England and Wales 1971-81. Br J Ind Med ;43: 158-164.

Hogstedt C, Jansson C, Hugosson M, *et al*.(2013) Cancer incidence in a cohort of Swedish chimney sweeps, 1958-2006. Am J Pub Health;103:1708-1714.

Howe HL, Wolfgang PE, Burnett WS, *et al*.(1989) Cancer incidence following exposure to drinking water with asbestos leachate. Publich Health Rep; 104:251-256.

Hu J, Mao Y, White K, *et al*.(2002) Renal cell carcinoma and occupational exposure to chemicals in Canada. Occup Med;52:157-164.

Huang J, Hisanaga N, Sakai K, *et al*.(1988) Asbestos fibers in human pulmonary and extrapulmonary tissues. Am J Ind Med 1988;14:331-339.

Karjalainen A, Pukkala E, Kauppinen *et al*.(1999)Incidence of cancer among Finnish patients with asbestos-related pulmonary or pleural fibrosis. Cancer Causes Control;10:51-57.

Kolonel LN, Yoshizawa CN, Hirohata T, *et al*.(1985) Cancer occurrence in shipyard workers exposed to asbestos in Hawaii. Cancer Res;45: 3924-3928.

Koskinen K, Pukkala E, Reijula K, *et al*.(2003) Incidence of cancer among the participants of the Finnish Asbestos Screening Campaign. Scand J Work Environ Health;29:64-70.

Krstev S, Stewart P, Rusiecki, *et al*.(2007) Mortality among shipyard Coast Guard workers: a retrospective cohort study. Occup Environ Med;64:651-658.

Kurumatani N, Natori Y, Mizutani R, *et al*.(1999) A historical cohort mortality study of workers exposed to asbestos in a refitting shipyard. Ind Health;37:9-17.

Levin JL, Rouk A, Shepherd S, *et al*.(2016) Tyler asbestos workers: A mortality update in a cohort exposed to amosite. J Toxicol Environ Health; 19:190-200.

Li L, Sun TD, Zhang X, *et al*.(2004)Cohort studies on cancer mortality among workers exposed only to chrysotile asbestos: a meta-analysis. Biomed Environ Sci;17:459-468.

Lotti M, Bergamo L, Murer B.(2010) Occupational toxicology of asbestos-related malignancies. Clin Toxicol; 48:485-496.

Luberto F, Ferrante D, Silvestri S, *et al*.(2019) Cumulative asbestos exposure and mortality from asbestos related diseases in a pooled analysis of 21 asbestos cement cohorts in Italy. Environmental Health; 18:71.

MacLeod JS, Harris MA, Tjepkema M, *et al*.(2017) Cancer Risks among Welders and Occasional Welders in a National Population-Based Cohort Study: Canadian Census Health and Environmental Cohort. Saf Health Work;8:258-266.

Magnani C, Ferrante D, Barone-Adesi F, *et al*.(2008) Cancer risk after cessation of asbestos exposure: a cohort study of Italian asbestos cement workers. Occup Environ Med;65:164-170.

Magnani C, Terracini B, Ivaldi C, *et al*.(1996)Mortality for cancer and other causes in asbestos cement workers at Casale Monferrato. Med Lav;87: 133-146.

Marant Micallef C, Shield KD, Baldi I, *et al*.(2018) Occupational exposures and cancer: a review of agents and relative risk estimates. Occup Environ Med;75:604-614.

Mariusdottir E, Ingimarsson JP, Jonsson E, *et al*.(2016) Occupation as a risk factor for renal cell cancer: a nationwide, prospective epidemiological study. Scand J Urol; 50:181-185.

Menegozzo S, comba P, Ferrante D, *et al*. (2011)Mortality study in an asbestos cement factory in Naples, Italy. Ann Ist Super Sanita;47:296-304.

Merlo DF, Bruzzone M, Bruzzi P, *et al*.(2018) Mortality among workers exposed to asbestos at the shipyard of Genoa, Italy: a 55 years follow-up. Environ Health; 17: 94.

Meurman LO, Pukkala E, Hakama M.(1994) Incidence of cancer among anthophyllite asbestos miners in Finland. Occup Environ Med;51: 421-425.

Michalek IM, Martinsen JI, Weiderpass E, *et al*.(2019) Occupation and risk of cancer of the renal pelvis in Nordic countries. BJU Int; 123:233-238.

Montanaro F, Ceppi M, Puntoni R, *et al*.(2004) Asbestos exposure and cancer mortality among petroleum refinery workers: a Poisson regression analysis of updated data. Arch Environ Health;59:188-193.

Neuberger M, Kundi M.(1990) Individual asbestos smoking and mortality - a cohort study in the asbestos cement industry. Br J Ind Med;47: 615-620.

Newhouse M, Berry G, Wagner JC.(1985) Mortality of factory workers in east London 1933-80. Br J of Ind Med;42:4-11.

Oddone E, Ferrante D, Tunesi S, *et al*.(2017)Mortality in asbestos cement workers in Pavia, Italy: A cohort study. Am J Ind Med; 60:852-866.

Ohlson C-G, Hogstedt C.(1985) Lung cancer among asbestos cement workers: a Swedish cohort study and a review. Br J Ind Med;42: 397-402.

Ohlson C-G, Klaesson B, Hogstedt C.(1984) Mortality among asbestos-exposed workers in a railroad workshop. Scand J Work Environ Health;10: 283-291.

Oksa P, Pukkala E, Karjalainen A, *et al*.(1997) Cancer incidence and mortality among Finnish asbestos sprayers and in asbestosis and silicosis patients. Am J Ind Med;31:693-698.

O’Reilly D, Reid J, Middleton R, *et al*.(1999) Asbestos related mortality in Northern Ireland: 1985-1994. J Public Health Med; 21:95-101.

Parent ME, Hua Y, Siemiatycki J.(2000) Occupational risk factors for renal cell carcinoma in Montreal. Am J Ind Med;38:609-618.

Partanen T, Heikkilä P, Hernberg S, *et al*.(1991) Renal cell cancer and occupational exposure to chemical agents. Scand J Work Environ Health; 17:231-239.

Pasetto R, Terracini B, Marsili D, *et al*.(2014) Occupational Burden of Asbestos-related Cancer in Argentina, Brazil, Colombia, and Mexico. Occupational. Ann Glob Health; 80:263-268.

Pesch B, Taeger D, Johnen G, *et al*.(2010) Cancer mortality in a surveillance cohort of German males formerly exposed to asbestos. Int J Hyg Environ Health; 213:44-51.

Peto J, Doll R, Howard SV, *et al*.(1977) A mortality study among workers in an English asbestos factory. Br J Ind Med; 34:169-173.

Pettinari A, Mengucci R, Belli S, *et al*.(1994)Mortality of workers employed at an asbestos cement manufacturing plant in Senigallia. Med Lav;85: 223-230.

Piolatto G, Negri E, La Vecchia C, *et al*.(1990) An update of cancer mortality among chrysotile asbestos miners in Balangero, northern Italy. Br J Ind Med;47:810-814.

Pira E, Pelucchi C, Buffoni L, *et al*.(2005) Cancer mortality in a cohort of asbestos textile workers. Br J Cancer;92:580-586.

Pira E, Pelucchi C, Piolatto PG, *et al*.(2009) Mortality from cancer and other causes in the Balangero cohort of chrysotile asbestos miners Occup Environ Med;66:805-809.

Pira E, Romano C, Violante FD, *et al*.(2016) Updated mortality study of a cohort of asbestos textile workers. Cancer Medicine; 5:2623-2628.

Pira E, Romano C, Donato F, *et al*.(2017) Mortality from cancer and other causes among Italian chrysotile asbestos miners. Occup Environ Med;74: 558-563.

Puntoni R, Vercelli M, Merlo F, *et al*.(1979) Mortality among shipyard workers in Genoa, Italy. Ann N Y Acad Sci; 330:353-377.

Puntoni R, Merlo F, Borsa L, *et al*.(2001) A Historical Cohort Mortality Study Among Shipyard Workers in Genoa, Italy. Am J Ind Med;40:363-370.

Raffn E, Villadsen E, Engholm G, *et al*.(1996) Lung cancer in asbestos cement workers in Denmark. BMJ ;53:399-402.

Reid A, Merler E, Peters S, *et al*.(2018) Migration and work in postwar Australia: mortality profile comparisons between Australian and Italian workers exposed to blue asbestos at Wittenoom. Occup Environ Med;75:29-36.

Repp K, Lorbeer R, Ittermann T, *et al*.(2015)Occupational exposure to asbestos is associated with increased mortality in men recruited for a population-based study in Germany. Int J Occup Med Environ Health;28:849-862.

Ribak J, Ribak G.(2008)Human health effects associated with the commercial use of grunerite asbestos (amosite): Paterson, NJ; Tyler, TX; Uxbridge, UK. Regul toxicol Pharmacol;52:S82-S90.

Ribak J, Seidman H, Selikoff IJ.(1989) Amosite mesothelioma in a cohort of asbestos workers. Scand J Work Environ Health;15:106-110.

Rösler JA, Woitowitz HJ.(1995) Recent data on cancer due to asbestos in Germany. Med Lav 1995; 86: 440-448.

Rubino GF, Piolatto G, Newhouse ML, *et al*.(1979) Mortality of chrysotile asbestos workers at the Balangero Mine, Northern Italy. Br J Ind Med;36:187-194.

Rusiecki J, Stewart P, Lee D, *et al*.(2018) Mortality among Coast Guard Shipyard workers: A retrospective cohort study of specific exposures. Arch Environ Occup Health; 73:4-18.

Schnatter AR, Wojcik NC, Jorgensen G.(2019) Mortality Update of a Cohort of Canadian Petroleum Workers. J Occup Environ Med; 61:225-238.

Schüz J, Schonfeld SJ, Kromhout H, *et al*.(2013) A retrospective cohort study of cancer mortality in employees of a Russian chrysotile asbestos mine and mills: study rationale and key features. Cancer Epidemiol;37:440-445.

Seidman H, Selikoff IJ, Gelb SK.(1986) Mortality experience of amosite asbestos factory workers: dose-response relationships 5 to 40 years after onset of short-term work exposure. Am J Ind Med;10:479-514.

Selikoff IJ, Seidman H.(1991) Asbestos-associated deaths among insulation workers in the United States and Canada, 1967-1987. Ann NY Acad Sci;643: 1-14.

Selikoff IJ, Lilis R, Nicholson WJ.(1979b) Asbestos disease in United States shipyards. Ann NY Acad Sci; 330: 295-311.

Sichletidis L, Chloros D, Spyratos D, *et al*.(2009) Mortality from occupational exposure to relatively pure chrysotile: a 39-year study. Respiration;78:63-68.

Smailyte G, Kurtinaitis J, Andersen A.(2004) Cancer mortality and morbidity among Lithuanian asbestos-cement producing workers. Scand J Work Environ Health;30:64-70.

Smartt P.(2004) Mortality, morbidity, and asbestosis in New Zealand: the hidden legacy of asbestos exposure. N Z Med J; 117:U1153.

Smith AH, Shearn VI, Wood R.(1989) Asbestos and kidney cancer: the evidence supports a causal association. Am J Ind Med; 16:159-166.

Sullivan PA.(2007) Vermiculite, respiratory disease, and asbestos exposure in Libby, Montana: update of a cohort mortality study. Environ Health Perspect;115:579-585.

Świątkowska B.(2019) The occurrence of asbestos-related diseases among former employees of asbestos processing plants in Poland. Med Pr; 70:723-731.

Szeszenia-Dąbrowska N, Świątkowska B, Sobala W, *et al*.(2015) Asbestos related diseases among workers of asbestos processing plants in relation to type of production and asbestos use. Med Pr; 66:1-9.

Szeszenia-Dąbrowska N, Wilczynska U, Szymczak W.(1988) Mortality among female workers in an asbestos factory in Poland. Pol J Occup Med;1:203-212.

Szeszenia-Dąbrowska N, Wilczynska U, Szymczak W.(1997) Cancer risk in asbestos-cement industry workers in Poland. Med Pr;48: 473-483.

Szeszenia-Dąbrowska N, Urszula W, Szymczak W, *et al*.(2002) Mortality study of workers compensated for asbestosis in Poland, 1970-1997. Int J Occup Med Environ Health;15:267-278.

Thomas HF, Benjamin IT, Elwood PC, *et al*.(1982)Further follow-up study of workers from an asbestos cement factory. Br J Ind Med 1982;39: 273-276.

Tsai SP, Waddell LC, Gilstrap EL, *et al*.(1996) Mortality among maintenance employees potentially exposed to asbestos in a refinery and petrochemical plant. Am J Ind Med;29:89-98.

Tulchinsky TH, Ginsberg GM, Shihab S, *et al.*(2001) Cancer in Ex-Asbestos Cement Workers in Israel, 1953–1992. Isr J Med Sci; 28:543-547.

Ulvestad B, Kjaerheim K, Martinsen JI, *et al*.(2002) Cancer incidence among workers in the asbestos-cement producing industry in Norway. Scand J Work Environ Health;28:411-417.

Van den Borre L, Deboosere P.(2015) Enduring health effects of asbestos use in Belgian industries: a record-linked cohort study of cause-specific mortality (2001–2009). BMJ Open;5: e007384.

Wang X, Lin S, Yano E, *et al*.(2012a)Mortality in a Chinese chrysotile miner cohort. Int Arch Occup Environ Health 2012; 85:405-412.

Wang X, Yano E, Qiu H*, et al*.(2012b) A 37-year observation of mortality in Chinese chrysotile asbestos workers. Thorax; 67:106-110.

Wang X, Lin S, Yu I, *et al*.(2013a) Cause-specific mortality in a Chinese chrysotile textile worker cohort. Cancer Sci; 104:245-249.

Wang X, Yano E, Lin S, *et al*.(2013b) Cancer mortality in Chinese chrysotile asbestos miners: exposure-response relationships. PLoS One;21:8:e71899.

Weiss W.(1977) Mortality of a cohort exposed to chrysotile asbestos. J Occup Med;19:737-740.

West GH, Sokas RK, Welch LS.(2019) Change in prevalence of asbestos-related disease among sheet metal workers 1986 to 2016. Am J Ind Med;62:609-615.

Wilczyńska U, Szymczak W, Szeszenia-Dąbrowska N.(2005) Mortality from malignant neoplasms among workers of an asbestos processing plant in Poland: results of prolonged observation. Int J Occup Med Environ Health;18:313-326.

Woitowitz HJ, Lange HJ, Beierl L, *et al*.(1986) Mortality rates in the Federal Republic of Germany following previous occupational exposure to asbestos dust. Int Arch Occup Environ Health;57:161-171.

Wu WT, Lin YJ, Li CY, *et al*.(2015) Cancer Attributable to Asbestos Exposure in Shipbreaking Workers: A Matched-Cohort Study. PLoS One;10: e0133128.

Yano E, Wang ZM, Wang XR, *et al*.(2001) Cancer mortality among workers exposed to amphibole-free chrysotile asbestos. Am J Epidemiol 2001;154:538-543.

Yano E.(2018) Adverse health effects of asbestos: solving mysteries regarding asbestos carcinogenicity based on follow-up survey of a Chinese factory. Environ Health Prev Med;23:35

Zhong F, Yano E, Wang ZM, *et al*.(2008)Cancer mortality and asbestosis among workers in an asbestos plant in Chongqing, China. Biomed Environ Sci;21:205-211.
