# Supplementary material for: Occupational Asbestos Exposure and Kidney Cancer: Systematic Review and Meta-analysis of Cohort Studies
Source: Ann Work Expo Health. 2020 Dec 26;65(3):255–65. doi: 10.1093/annweh/wxaa114 (PMC8062011; doi:10.1093/annweh/wxaa114)
Supplement: wxaa114_suppl_Supplementary_Tables_S1_S2_S3_Review [file wxaa114_suppl_supplementary_tables_s1_s2_s3_review.docx]

**Title:** Occupational asbestos exposure and kidney cancer: Systematic Review and Meta-analysis of Cohort Studies.

**Authors:** Chris CY Pang, Kevin Phan, Md Nazmul Karim, Afsana Afroz, Matthew Winter, Deborah C Glass

**Supplementary Table S1. Characteristics of mortality studies included in the meta-analysis.**

| **Study** | **Country** | **NOS rating** | **Industry type** | **Asbestos type** | **Asbestos exposure (Fibres/ml)** | **Cohort size** | **Period of employment** | **Follow-up period** | **Total person years follow-up** | **Lung Cancer SMR** | **Observed/ Expected kidney cancer deaths** | **Kidney Cancer SMR (95%CI)** |
| --- | --- | --- | --- | --- | --- | --- | --- | --- | --- | --- | --- | --- |
| Ferrante  *et al*.2017 | Italy | Good | Various | Undefined | NA | 46060 | <1949-2010 | 1970-2010 | 1430713 | 1.26 | 157/160.7 | 0.98(0.83-1.14) |
| Pira *et al*. 2017 | Italy | Good | Mining | Chrysotile | 1950: 37; 1971-1976: 5 | 1056 | 1930-1990 | 1946-2014 | 37471 | 1.16 | 2/3.2 | 0.62(0.07-2.23) |
| Levin *et al*. 2016 | USA | Good | Insulation | Amphiboles | 1967-1971: 2.5-163.5 | 1130 | 1954-1972 | 1960-2011 | NA | 2.44 | 1/2.7 | 0.37(0.01-2.06) |
| Pira *et al*. 2016 | Italy | Fair | Textile | Chrysotile | Late 1960s:100; Early 1970s: 5–25; Late 1970s: up to 2 | 1977 | 1946-1984 | 1946-2013 | 74126 | 2.96 | 4/4.12 | 0.97(0.26-2.49) |
| Van den Borre and Deboosere. 2015 | Belgium | Good | Various | Undefined | NA | 2056 | NA | 2001-2009 | NA | 1.42 | 3/1.37 | 2.19(0.56-5.96) |
| Wang *et al*. 2013 | China | Good | Textile | Chrysotile | 2002: 6-18 | 865 | NA | 1972-2008 | 27475 | NA | 1/0.45 | 2.22(0.11-10.96) |
| Pesch *et al*. 2010 | Germany | Poor | Various | Undefined | NA | 576 | 1936-1991 | 1993-2007 | 6642.8 | 0.39 | 1/2.7 | 0.37(0.01-2.07) |
| Harding *et al*. 2009 | UK | Good | Various | Undefined | NA | 98117 | <1920-2005 | 1971-2005 | 1779580 | 1.87 | 114/74.5 | 1.53(1.26-1.83) |
| Dement *et al*. 2009 | USA | Good | Sheet Metal Workers | Undefined | NA | 17345 | <1966-1986 | 1986-2004 | 207442 | 1.02 | 34/40.75 | 0.83(0.58-1.17) |
| Krstev *et al*. 2007 | USA | Good | Shipyard | Undefined | NA | 4702 | 1950-1964 | 1964-2001 | 150049 | 1.27 | 10/14.8 | 0.68(0.34-1.2) |
| Wilczyńska *et al*. 2005 | Poland | Good | Asbestos Factory | Undefined | NA | 4187 | 1945-1980 | 1980-1999 | 118742 | 1.37 | 5/7.79 | 0.64(0.24-1.42) |
| Szeszenia-Dąbrowska  *et al*. 2002 | Poland | Fair | Various | Undefined | NA | 1397 | NA | 1970-1999 | NA | 2.06 | 3/2.53 | 1.19(0.30-3.23) |
| Puntoni *et al*. 2001 | Italy | Good | Shipyard | Undefined | NA | 3984 | 1960-1981 | 1960-1996 | 79576 | 1.77 | 10/10.4 | 0.96(0.46-1.76) |
| Berry *et al*. 2000 | UK | Good | Asbestos Factory | Undefined | NA | 5000 | 1933-1980 | 1933-1980 | NA | 3.01 | 2/3.64 | 0.55(0.07-1.99) |
| Szeszenia-Dąbrowska  *et al*. 1997 | Poland | Good | Cement | Undefined | 10 | 4712 | 1945-1990 | 1945-1990 | NA | NA | 2/3.61 | 0.55(0.07-2.00) |
| Germani *et al*. 1996 | Italy | Good | Various | Undefined | NA | 3417 | NA | 1980-1990 | NA | NA | 3/4.1 | 0.73(0.15-2.14) |
| Rösler and Woitowitz. 1995 | Germany | Good | Various | Undefined | NA | 3988 | 1913-1968 | 1977-1988 | NA | 1.48 | 3/5.66 | 0.53(0.11-1.55) |
| Dement *et al*. 1994 | USA | Good | Textile | Chrysotile | 1940-1990: 4.9 - 12 | 3022 | 1930-1965 | 1940-1990 | 115874 | 1.97 | 4/4.59 | 0.87(0.24-2.23) |

| **Study** | **Country** | **NOS rating** | **Industry type** | **Asbestos type** | **Asbestos exposure (Fibres/ml)** | **Cohort size** | **Period of employment** | **Follow-up period** | **Total person years follow-up** | **Lung Cancer SMR** | **Observed/ Expected kidney cancer deaths** | **Kidney Cancer SMR (95%CI)** |
| --- | --- | --- | --- | --- | --- | --- | --- | --- | --- | --- | --- | --- |
| Meurman  *et al*. 1994 | Finland | Good | Mining | Amphiboles | Late 1960s: mean 50 | 903 | 1918-1995 | 1953-1995 | 25407 | 2.88 | 1/1.48 | 0.68(0.02-3.76) |
| McDonald  *et al*. 1993 | Canada | Good | Mining | Chrysotile | NA | 5335 | 1904-1966 | 1976-1988 | NA | 1.4 | 13/13 | 1.00(0.53-1.71) |
| Selikoff and Seidman.  1991 | USA and Canada | Fair | Insulation | Undefined | 1940s-1950s: 15-20; early 1960s: 10; late 1960s-1970s: 5 | 17800 | 1953-1986 | 1967-1986 | 301592.6 | 4.35 | 32/18.87 | 1.70(1.16-2.39) |
| Neuberger and Kundi. 1990 | Austria | Good | Cement | Chrysotile | Median - 12 | 2816 | 1950-1981 | 1950-1986 | 51218 | 1.72 | 1/1.8 | 0.56(0.01-3.10) |
| Albin *et al*. 1990 | Sweden | Good | Cement | Chrysotile | 1956-1977: Median 1.2 | 2567 | 1907-1977 | 1952-1995 | 67077.3 | 1.8 | 7/7.88 | 0.85(0.36-1.83) |
| Ribak *et al*. 1989 | USA | Poor | Asbestos Factory | Amphiboles | Average: 50 | 820 | 1941-1945 | 1941-1988 | 18190 | NA | 3/1.7 | 1.76(0.36-5.16) |
| Armstrong  *et al*. 1988 | Australia | Good | Mining | Amphiboles | 1966: 20 to 100 | 6917 | 1943-1966 | 1943-1980 | 100178 | 2.64 | 7/11.6 | 0.60(0.24-1.24) |
| Hughes *et al*. 1987 | USA | Good | Cement | Chrysotile | 1952-1969: Mean -10.6 | 6931 | 1940-1982 | 1940-1982 | NA | 1.34 | 7/5.3 | 1.32(0.53-2.72) |
| Enterline *et al*. 1987 | USA | Good | Asbestos Factory | Undefined | NA | 1074 | 1941-1967 | 1941-1980 | 11640.3 | 2.71 | 7/2.54 | 2.76(1.11-5.68) |
| Gardner *et al*. 1986 | UK | Good | Cement | Chrysotile | 1968-1982: 0.2-9.0 | 2173 | 1941-1983 | 1941-1984 | NA | 0.98 | 0/2.14 | 0.0(0.0-1.72) |
| Ohlson and Hogstedt. 1985 | Sweden | Good | Cement | Chrysotile | 1970-1976: 1-2 | 1176 | 1943-1976 | 1951-1982 | 26931 | 1.23 | 3/3.2 | 0.94(0.19-2.74) |
| Peto *et al*. 1985 | UK | Good | Textile | Chrysotile | NA | 3639 | <1933-1983 | 1933-1983 | NA | 1.45 | 1/4.47 | 0.22(0.01-1.10) |
| Kolonel *et al*. 1985 | USA | Good | Shipyard | Undefined | NA | 7971 | 1934-1969 | 1950-1982 | 106742 | 1.09 | 9/9.51 | 0.95(0.43-1.8) |
| Acheson *et al*. 1984 | UK | Good | Insulation | Amphiboles | Late 1960s: 30 | 4820 | 1947-1979 | 1947-1979 | 68922 | 2.1 | 4/3.42 | 1.17(0.32-3.00) |
| Ohlson *et al*. 1984 | Sweden | Good | Rail | Undefined | 1970s:3-20 | 3297 | 1902-1980 | 1951-1980 | NA | 1.16 | 10/10.6 | 0.94(0.45-1.73) |
| Acheson *et al*. 1982 | UK | Good | Gas Mask Factory | Chrysotile | NA | 570 | 1939-1980 | 1951-1980 | 14324 | 1.45 | 0/0.47 | 0.0(0-7.83) |
| Thomas *et al*. 1982 | UK | Good | Cement | Chrysotile | NA | 1592 | 1936-1977 | 1936-1977 | 38484 | NA | 1/2.0 | 0.50(0.01-2.79) |
| Acheson *et al*. 1982 | UK | Good | Gas Mask Factory | Amphiboles | NA | 757 | 1939-1980 | 1951-1980 | 18781 | 2.41 | 0/0.63 | 0.0(0-5.86) |
| Selikoff *et al*. 1979(a) | USA | Good | Insulation | Undefined | 1940s-1950s: 15-20 early; 1960s: 10; late 1960s-1970s: 5 | 632 | 1943-1962 | 1943-1976 | 13925 | 7 | 1/0.2 | 5.00(0.13-27.86) |
| Selikoff *et al*. 1979(b) | USA | Good | Insulation | Undefined | 1940s-1950s: 15-20 early; 1960s: 10; late 1960s-1970s: 5 | 833 | 1943-1976 | 1943-1976 | 18801 | 3.68 | 2/1.33 | 1.50(0.18-5.43) |

**Supplementary Table S2. Characteristics of incidence studies included in the meta-analysis.**

| **Study** | **Country** | **NOS rating** | **Industry type** | **Asbestos type** | **Asbestos exposure (Fibres/ml)** | **Cohort size** | **Period of employment** | **Follow-up period** | **Total person years follow-up** | **Lung Cancer SIR** | **Observed/Expected Kidney cancer cases** | **Kidney Cancer SIR (95%CI)** |
| --- | --- | --- | --- | --- | --- | --- | --- | --- | --- | --- | --- | --- |
| Barbiero  *et al*. 2018 | Italy | Good | Various | Undefined | NA | 2488 | 1974-1994 | 1995-2009 | 19514 | 1.61 | 11/13.36 | 0.82(0.41-1.47) |
| Wu *et al*. 2015 | Taiwan | Good | Shipyard | Undefined | 1987: 0.02-0.208 | 4427 | 1975-2008 | 1985-2008 | 109932 | 2.71 | 4/19^a^ | 1.28(0.43-3.85)^b^ |
| Koskinen  *et al*. 2003 | Finland | Good | Various | Undefined | NA | 24215 | 1950s-1998 | 1990-1998 | 174582 | 1.15 | 67/63.36 | 1.06(0.83-1.34) |
| Ulvestad  *et al*. 2002 | Norway | Good | Cement | Chrysotile | 1950: 50 to 1000; 1954: 50 to 100; 1964: 100 to 1900; 1972 to 1978: 13, After 1973: 5 | 541 | 1942-1968 | 1953-1999 | 17264 | 3.1 | 4/3.08 | 1.30(0.40-3.40) |
| Tulchinsky *et al*. 1999 | Israel | Good | Cement | Chrysotile | Pre-1978: 0.3-1.5; Post-1978: 0.1-0.4 | 3057 | 1953-1992 | 1953-1992 | NA | 1.35 | 5/4.8 | 1.04(0.13-1.95) |
| Raffn *et al*. 1996 | Denmark | Good | Cement | Amphiboles | 1948: 50 - 800; 1957:10-100; 1973: >2.0 f/ml in 41% of samples. | 8580 | 1928-1984 | 1943-1990 | 155767 | 1.7 | 22/23.82 | 0.92(0.58-1.40) |
| Englund. 1995 | Sweden | Good | Insulation | Undefined | NA | 1690 | 1967-1991 | 1967-1991 | NA | 1.31 | 8/5.7 | 1.40(0.65-2.67) |
| Meurman  *et al*. 1994 | Finland | Good | Mining | Amphiboles | Late 1960s: mean 50 | 903 | 1918-1995 | 1953-1995 | 25407 | 2.88 | 6/3.24 | 1.85(0.68-4.03) |
| Sandén  *et al*. 1992 | Sweden | Good | Shipyard | Amphiboles | Pre-1964: >2 f/ml in 44% of measurements. | 3893 | 1950-1972 | 1977-1987 | 35155.8 | 0.85 | 6/12.4 | 0.48(0.18-1.10) |
| Albin *et al*. 1990 | Sweden | Good | Cement | Chrysotile | 1956-1977: Median 1.2 | 2567 | 1907-1977 | 1958-1994 | 67077.3 | 1.8 | 10/11.33 | 0.84(0.40-1.54) |
| Armstrong *et al*. 1988 | Australia | Good | Mining | Amphiboles | 1966: 20 to 100 | 6917 | 1943-1966 | 1943-1980 | 100178 | 2.64 | 17/16.5 | 1.03(0.60-1.65) |

^a^ Incident cases among cohort/ Incident cases among controls.

^b^ Hazard ratio.

**Supplementary Table S3. Table summarising the reasons for the exclusion of studies.**

| **Study** | **Geographic location** | **Reason for exclusion** | **Industry type/study group** | **Asbestos type** |
| --- | --- | --- | --- | --- |
| Weiss *et al*. 1977 | USA | No kidney cancer outcome | Asbestos plant | Chrysotile |
| Elmes *et al*. 1977 | Ireland | No kidney cancer outcome | Insulation | Undefined |
| Peto *et al*. 1977 | UK | No kidney cancer outcome | Asbestos factory | Undefined |
| Blot *et al*. 1979 | USA | Non-occupational asbestos exposure | N/A | Undefined |
| Rubino *et al*. 1979 | Italy | Updated by Ferrante 2017 | Mining | Chrysotile |
| Puntoni *et al*. 1979 | Italy | Updated by Puntoni 2001 | Shipyard workers | Undefined |
| Beaumont *et al*. 1980 | USA | Multiple exposures, asbestos not the predominant exposure | Various industries | Undefined |
| Dement *et al*. 1983 | USA | Updated by Dement 1994 | Textile | Chrysotile |
| Alies-Patin *et al*. 1985 | France | No kidney cancer outcome | Asbestos cement | Chrysotile |
| Newhouse *et al*. 1985 | UK | Updated by Berry 2000 | Asbestos factory | Mixed |
| Hodgson and Jones 1986 | UK | Updated by Harding 2009 | Various industries | Undefined |
| Woitowitz *et al*. 1986 | Germany | No kidney cancer outcome. Reported as "urogenital cancer". No description of kidney cancer specifically | Various industries | Undefined |
| Seidman *et al*. 1986 | USA | Updated by Ribak 1989 | Asbestos factory | Amphibole |
| Huang *et al*. 1988 | Japan | Autopsy study investigating concentration of asbestos in extrapulmonary tissue | Autopsy studies | Mixed |
| Szeszenia-Dąbrowska *et al*. 1988 | Poland | Updated by Wilczyńska 2005 | Asbestos plant | Undefined |
| Finkelstein 1989 | Canada | No kidney cancer outcome | Friction material manufacturer | Chrysotile |
| Howe *et al*. 1989 | USA | Non-occupational asbestos exposure | N/A | Undefined |
| Smith *et al*. 1989 | USA | Review paper | Various studies | Undefined |
| Piolatto *et al*. 1990 | Italy | Updated by Ferrante 2017 | Mining | Chrysotile |
| Dement 1991 | USA | Case-control study. No kidney cancer data | Textile | Chrysotile |
| Partanen *et al*. 1991 | Finland | Multiple exposures, asbestos not the predominant exposure | Various industries, including those from industries where asbestos is not the predominant exposure. | Undefined |
| Cheng *et al*. 1992 | China | No kidney cancer outcome | Asbestos textile, friction material, cement manufacturing. | Chrysotile |
| Gibbs *et al*. 1994 | UK | Investigated lung cancer and mesothelioma | Asbestos factory | Amphibole |
| Berry 1994 | UK | Review paper | Friction material manufacturer | Chrysotile |
| Giaroli *et al*. 1994 | Italy | Updated by Ferrante 2017 | Asbestos cement workers | Mixed |
| Pettinari *et al*. 1994 | Italy | Updated by Ferrante 2017 | Asbestos plant | Mixed |
| Delahunt *et al*. 1995 | New Zealand | Case-control study. No asbestos exposure data | Various industries | Undefined |
| Tsai *et al*. 1996 | USA | Multiple exposures, asbestos not the predominant exposure | Petrochemical plant | Undefined |
| Magnani *et al*. 1996 | Italy | Updated by Ferrante 2017 | Asbestos cement | Mixed |
| Oksa *et al*. 1997 | Finland | Updated by Koskinen 2003 | Various asbestos related industries | Mixed |
| Kurumatani *et al*. 1999 | Japan | No kidney cancer outcome | Shipyard workers | Undefined |
| O'Reilly *et al*. 1999 | Ireland | Reported pleural cancer, peritoneal cancer and asbestosis. Other cancers were not the focus of research | Various industries | Undefined |
| Germani *et al*.  1999 | Italy | Study population more representative in Germani 1996 | Women compensated for asbestosis | Mixed |
| Battista *et al*.  1999 | Italy | Updated by Ferrante 2017 | Railway | Undefined |
| Karjalainen *et al*. 1999 | Finland | Updated by Koskinen 2003 | Patients with asbestos-related pulmonary or pleural fibrosis. | Undefined |
| Parent *et al*.  2000 | Canada | Multiple exposures, asbestos not the predominant exposure. Case-control study | Various industries | Undefined |
| Yano *et al*. 2001 | China | No kidney cancer outcome | Asbestos plant | Chrysotile |

| **Study** | **Geographic location** | **Reason for exclusion** | **Industry type/study group** | **Asbestos type** |
| --- | --- | --- | --- | --- |
| Hu *et al*. 2002 | Canada | Multiple exposures, asbestos not the predominant exposure. Case-control study | Various industries | Undefined |
| Smartt 2004 | New Zealand | Investigated asbestosis as outcome | Various industries | Undefined |
| Li *et al*. 2004 | China | Meta-analysis of chrysotile exposure | Various industries | Chrysotile |
| Montanaro *et al*. 2004 | Italy | Multiple exposures, asbestos not the predominant exposure | Petroleum refinery | Undefined |
| Smailyte *et al*. 2004 | Lithuania | No kidney cancer outcome | Asbestos cement | Chrysotile |
| Golka *et al*.  2004 | Germany | Review paper | Various industries | Undefined |
| Ji *et al*. 2005 | Sweden | No asbestos exposure data, based on occupations which included jobs that do not have asbestos as the predominant exposure | Various industries | Undefined |
| Pira *et al*.  2005 | Italy | Updated by Pira 2016 | Textile | Chrysotile |
| Gun *et al*.  2006 | Australia | Multiple exposures, asbestos not the predominant exposure | Petroleum refinery | Undefined |
| Sullivan 2007 | USA | No kidney cancer outcome | Mining | Mixed |
| Ferrante *et al*. 2007 | Italy | Non-occupational asbestos exposure among wives of asbestos workers | n/a | Mixed |
| Hein *et al*.  2007 | USA | Reported as "urinary organs" cancers, and no description of kidney cancer specifically | Textile | Chrysotile |
| Ribak *et al*.  2008 | USA | Reported lung cancer and mesothelioma outcomes | Asbestos plant | Amphibole |
| Zhong *et al*. 2008 | China | No kidney cancer outcome | Asbestos plant | Chrysotile |
| Magnani *et al*. 2008 | Italy | Updated by Ferrante 2017 | Asbestos cement | Mixed |
| Sichletidis *et al*. 2009 | Greece | No kidney cancer outcome | Asbestos cement | Chrysotile |
| Pira *et al*.  2009 | Italy | Updated by Pira 2017 | Mining | Chrysotile |
| Heck *et al*.  2010 | Europe | Multiple exposures, asbestos not the predominant exposure. Case-control study | Various industries | Undefined |
| Lotti *et al*.  2010 | Italy | Review paper | Various industries | Undefined |
| Clin *et al*.  2011 | France | Combined bladder and kidney cancer. No significant association with asbestos exposure | Various industries | Undefined |
| Courtice *et al*. 2011 | Bangladesh | No kidney cancer outcome | Ship breakers | Undefined |
| Menegozzo *et al*. 2011 | Italy | Updated by Ferrante 2017 | Asbestos cement | Mixed |
| Bunderson-Schelvan *et al*. 2011 | USA | Review paper | Various industries | Undefined |
| Wang *et al*.  2012 | China | No kidney cancer outcome | Asbestos factory | Chrysotile |
| Wang *et al*.  2012 | China | No kidney cancer outcome | Mining | Chrysotile |
| Giordano *et al*. 2012 | Italy | No kidney cancer outcome | Asbestos cement | Undefined |
| Du *et al*. 2012 | China | No kidney cancer outcome | Mining | Chrysotile |
| Schüz *et al*.  2013 | Russia | Described study rationale and protocol. No outcomes reported | Mining | Chrysotile |
| Hogstedt *et al*. 2013 | Sweden | Multiple exposures, asbestos not the predominant exposure | Chimney Sweepers | Undefined |
| Wang *et al*.  2013 | China | No kidney cancer outcome | Mining | Chrysotile |
| Pasetto *et al*. 2014 | Mexico and South America | Reported lung, larynx, and ovary outcomes | Various industries, including those from industries where asbestos is not the predominant exposure. | Undefined |
| Repp *et al*.  2015 | Germany | No kidney cancer outcome | Various industries/ administered questionnaire | Undefined |
| Szeszenia-Dąbrowska *et al*. 2015 | Poland | Reported mesothelioma and asbestosis | Asbestos plant | Mixed |

| **Study** | **Geographic location** | **Reason for exclusion** | **Industry type/study group** | **Asbestos type** |
| --- | --- | --- | --- | --- |
| Mariusdottir *et al*. 2016 | Iceland | Categorised exposure according to job roles. Asbestos not mentioned | Various industries, including those from industries where asbestos is not the predominant exposure. | Undefined |
| MacLeod *et al*. 2017 | Canada | Multiple exposures, asbestos not the predominant exposure | Welders | Undefined |
| Oddone *et al*. 2017 | Italy | Same cohort studied in Ferrante 2017 | Asbestos cement | Mixed |
| Peters *et al*. 2018 | Canada | Case-control study | Various industries | Undefined |
| Petersen *et al*. 2018 | Denmark | Multiple exposures, asbestos not the predominant exposure | Sea farers | Undefined |
| Rusiecki *et al*. 2018 | USA | Multiple exposures, asbestos not the predominant exposure | Shipyard workers | Undefined |
| DeBono *et al*. 2018 | USA | Multiple exposures, asbestos not the predominant exposure | Automotive manufacturer | Undefined |
| Reid *et al*.  2018 | Australia | No kidney cancer outcome | Wittenoom miners | Amphibole |
| Merlo *et al*. 2018 | Italy | Reported as "urogenital system diseases", and no description of kidney cancer specifically | Shipyard workers | Undefined |
| Yano 2018 | China | Review paper | Asbestos factory | Mixed |
| Marant Micallef *et al*. 2018 | France | Review paper | Various industries | Undefined |
| Michalek *et al*. 2019 | Nordic countries | Case-control study | Various industries | Undefined |
| Luberto *et al*. 2019 | Italy | Ferrante 2017 was more representative of the exposed cohort | Asbestos cement | Mixed |
| Schnatter *et al*. 2019 | Canada | Multiple exposures, asbestos not the predominant exposure | Petroleum industry | Undefined |
| Finkelstein *et al*. 2019 | USA | Reported mesothelioma outcome | Friction material manufacturer | Chrysotile |
| Barone-Adesi *et al*. 2019 | Italy | Reported pleural and peritoneal cancer | Various industries | Undefined |
| Świątkowska *et al*. 2019 | Poland | Reported pleural disease and asbestosis outcomes | Various asbestos related industries | Undefined |
| West *et al*.  2019 | USA | Reported respiratory diseases outcomes: pleural disease, lung parenchymal disease | Various industries | Undefined |

**Supplementary Table S4. Table summarising the quality of exposure assessment criteria.**

| **Study** | **Documentation^a^** | **Cumulative exposure^b^** | ***Conversion***  ***factor^c^*** | ***Coverage of***  ***exposure^d^*** | ***Job histories^e^*** |
| --- | --- | --- | --- | --- | --- |
| ***Mortality studies*** |  |  |  |  |  |
| Berry *et al*. (2000) | Yes | No | No | Yes | Yes |
| McDonald *et al*.(1993) | Yes | Yes | Yes | Yes | Yes |
| Hughes *et al.* (1987) | Yes | No | Yes | Yes | Yes |
| Peto *et al.* (1985) | Yes | Yes | Yes | Yes | Yes |
| Selikoff *et al*.(1979) | Yes | No | No | No | No |
|  |  |  |  |  |  |
| ***Incidence study*** |  |  |  |  |  |
| Sandén *et al*.(1992) | Yes | No | No | Yes | Yes |

The criteria were met if the studies satisfied the following requirements according to Lenters *et al*.(2011);

^a^ Documentation: The authors sufficiently described the exposure assessment in terms of number of dust, variability in exposure categories, details about analytical procedures, and so forth.

^b^ Cumulative exposure: There was an adequate contrast in exposure within a cohort study, between the average values of exposure within the highest versus lowest cumulative exposure categories.

^c^ Conversion factor: The studies were classified according to the use of internal or external measurement conversion factors to account for changes over time in analytical and measurement techniques.

^d^ Coverage of exposure: Percentage of the accumulated work history years temporally covered by exposure measurement data was more than 30%.

^e^ Job histories: The job history information was sufficiently complete and detailed.

‘Yes’ indicates that the criterion was met. ‘No’ indicates that the criterion was not met.
