# Supplementary material for: Occupational Asbestos Exposure and Kidney Cancer: Systematic Review and Meta-analysis of Cohort Studies
Source: Ann Work Expo Health. 2020 Dec 26;65(3):255–65. doi: 10.1093/annweh/wxaa114 (PMC8062011; doi:10.1093/annweh/wxaa114)

**Title:** Occupational asbestos exposure and kidney cancer: Systematic Review and meta-analysis of Cohort Studies.

**Authors:** Chris CY Pang, Kevin Phan, Md Nazmul Karim, Afsana Afroz, Matthew Winter, Deborah C Glass

**Supplementary Figure S5**

SMR estimates and 95%CIs of kidney cancer associated with occupational asbestos exposure, for the four mortality studies with adequate exposure assessment data specific to kidney cancer outcomes. Weights are from fixed-effects analysis. Study specific SMRs are shown as squares, with the horizontal lines representing the 95% CIs for the study specific SMRs. The area of the squares represents weight of the individual study. The pooled-SMR is shown as a diamond. The middle of the diamond corresponds to the pooled-SMR, and the width of the diamond represents the 95%CI. The vertical dashed line provides a visual comparison of the pooled-SMR with the corresponding study specific SMRs. Abbreviations: *Q*, Cochran Q test, *p*, *p*-value for Cochran Q test; *I^2^*, *I^2^* statistic.

**
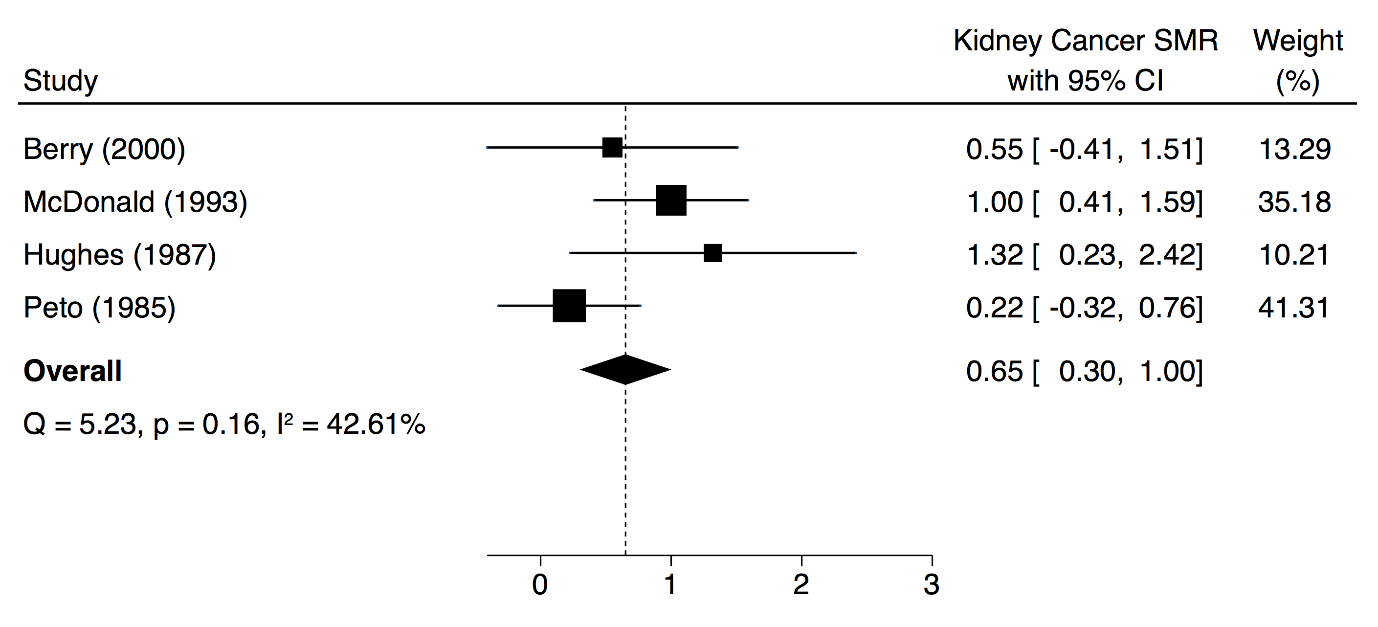
**

**Supplementary Figure S6**

Sensitivity analysis by omission of individual mortality studies in turn, using fixed-effects model-based meta-analysis. Rows represent the results of meta-analysis of all studies except the omitted study named in that row, with the resulting pooled-SMR shown as diamonds, with the size of the boxes being inversely proportional to the pooled-SMR variance. Solid horizontal lines represent the 95% CIs for the pooled-SMR excluding the omitted study named in that row. The solid vertical line represents the pooled-SMR obtained using all studies.


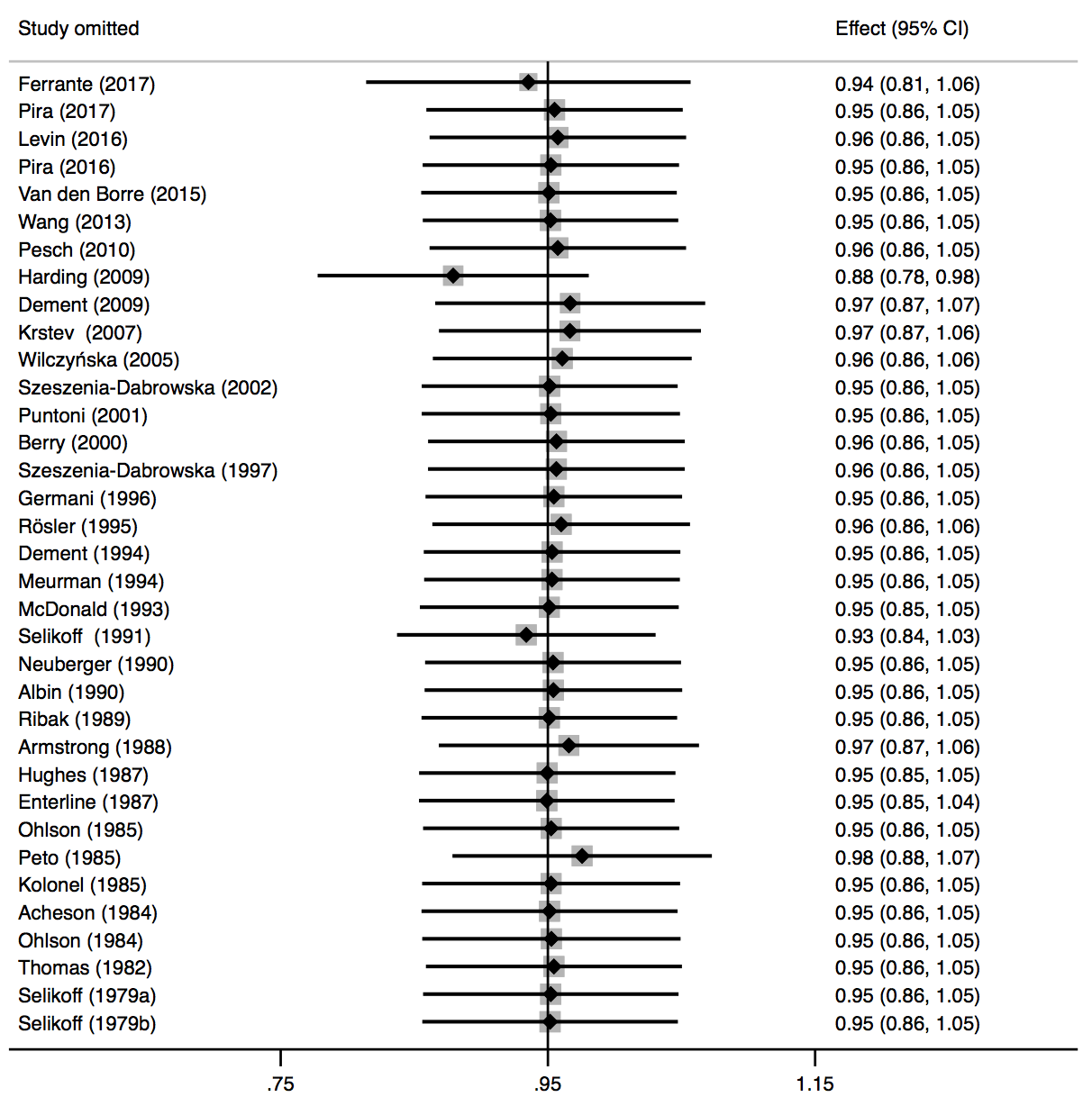


**Supplementary Figure S7**

Sensitivity analysis by omission of individual incidence studies in turn, using fixed-effects model-based meta-analysis. Rows represent the results of meta-analysis of all studies except the omitted study named in that row, with the resulting pooled-SIR shown as diamonds, with the size of the boxes being inversely proportional to the pooled-SIR variance. Solid horizontal lines represent the 95% CIs for the pooled-SIR excluding the omitted study named in that row. The solid vertical line represents the pooled-SIR obtained using all studies.


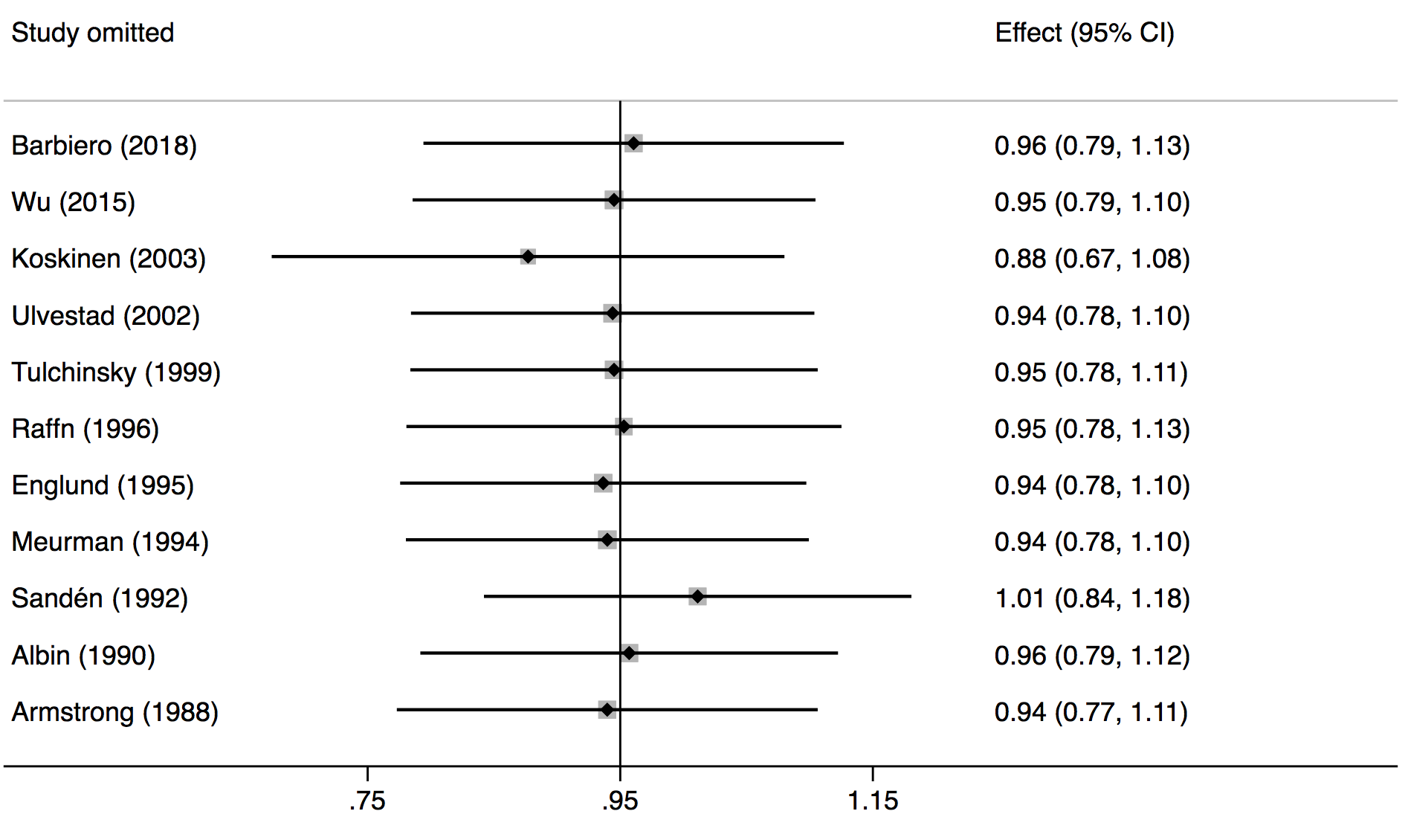

Supplement: wxaa114_suppl_Supplementary_Figures_S5_S6_S7 [file wxaa114_suppl_supplementary_figures_s5_s6_s7.docx]
